# On a Subclass of Meromorphic Close-to-Convex Functions

**DOI:** 10.1155/2014/806168

**Published:** 2014-08-28

**Authors:** Ming-Liang Li, Lei Shi, Zhi-Gang Wang

**Affiliations:** ^1^School of Mathematics and Statistics, Central South University, Changsha, Hunan 410075, China; ^2^School of Mathematics and Computing Science, Hunan First Normal University, Changsha, Hunan 410205, China; ^3^School of Mathematics and Statistics, Anyang Normal University, Anyang, Henan 455000, China

## Abstract

The main purpose of this paper is to introduce and investigate a certain subclass of meromorphic close-to-convex functions. Such results as coefficient inequalities, convolution property, inclusion relationship, distortion property, and radius of meromorphic convexity are derived.

## 1. Introduction and Preliminaries

Let Σ denote the class of functions *f* of the form

(1) $$\begin{array}{c} f\left(z\right)=\frac{1}{z}+\overset{\infty }{\underset{n=1}{\sum }}a_{n}z^{n}, \end{array}$$

which are* analytic* in the* punctured* open unit disk

(2) $$\begin{array}{c} U^{*}∶=\left\{z:z\in C\;\;0<\left|z\right|<1\right\}=:U\setminus \left\{0\right\}. \end{array}$$

For two functions *f* and *g*, analytic in *U*, we say that the function *f* is subordinate to *g* in *U* and write

(3) $$\begin{array}{c} f\left(z\right)≺g\left(z\right)\;\left(z\in U\right), \end{array}$$

if there exists a Schwarz function *ω*, which is analytic in *U* with

(4) $$\begin{array}{c} \omega \left(0\right)=0,\;\;\left|\omega \left(z\right)\right|<1\;\;\left(z\in U\right), \end{array}$$

such that

(5) $$\begin{array}{c} f\left(z\right)=g\left(\omega \left(z\right)\right)\;\left(z\in U\right). \end{array}$$

Indeed, it is known that

(6) f(z)≺g(z) (z∈U)⟹f(0)=g(0),      f(U)⊂g(U).

Furthermore, if the function *g* is univalent in *U*, then we have the following equivalence:

(7) f(z)≺g(z) (z∈U)⟺f(0)=g(0),        f(U)⊂g(U).

Let *f*, *g* ∈ Σ, where *f* is given by (1) and *g* is defined by

(8) $$\begin{array}{c} g\left(z\right)=\frac{1}{z}+\overset{\infty }{\underset{n=1}{\sum }}b_{n}z^{n}. \end{array}$$

Then the Hadamard product (or convolution) *f*∗*g* of the functions *f* and *g* is defined by

(9) $$\begin{array}{c} \left(f*g\right)\left(z\right)∶=\frac{1}{z}+\overset{\infty }{\underset{n=1}{\sum }}a_{n}b_{n}z^{n}=:\left(g*f\right)\left(z\right). \end{array}$$

A function *f* ∈ Σ is said to be in the class MS* of* meromorphic starlike functions* if it satisfies the inequality

(10) $$\begin{array}{c} R\left(\frac{zf^{\prime }\left(z\right)}{f\left(z\right)}\right)<0\;\left(z\in U\right). \end{array}$$

A function *f* ∈ Σ is said to be in the class *MK* of* meromorphic convex functions* if it satisfies the inequality

(11) $$\begin{array}{c} R\left(1+\frac{zf^{\prime \prime }\left(z\right)}{f^{\prime }\left(z\right)}\right)<0\;\left(z\in U\right). \end{array}$$

Moreover, a function *f* ∈ Σ is said to be in the class *MC* of* meromorphic close-to-convex functions* if it satisfies the condition

(12) $$\begin{array}{c} R\left(\frac{zf^{\prime }\left(z\right)}{g\left(z\right)}\right)<0\;\left(z\in U;\;g\in MS^{*}\right). \end{array}$$

Let

(13) $$\begin{array}{c} f\left(z\right)=z+a_{2}z^{2}+a_{3}z^{3}+\cdots \end{array}$$

be analytic in *U*. If there exists a function *g* ∈ *K* such that

(14) |(zf′(z)/g(z))−1(zf′(z)/g(z))+(1−2α)|<β(z∈U; 0≦α<1; 0<β≦1),

then we say that *f* ∈ *C*(*α*, *β*), where *K* denotes the usual class of convex functions. The function class *C*(*α*, *β*) was introduced and studied recently by Peng [1] (see also Peng and Han [2], Selvaraj [3], Gao and Zhou [4], Kowalczyk and Leś-Bomba [5], and Xu et al. [6]).

Motivated essentially by the above mentioned function class *C*(*α*, *β*), we now introduce and investigate the following class of meromorphic close-to-convex functions.

**Definition 1 .**

A function *f* ∈ Σ is said to be in the class *MC*(*α*, *β*) if it satisfies the inequality

(15) $$\begin{array}{c} \left|\frac{\left(zf^{\prime }\left(z\right)/g\left(z\right)\right)+1}{\left(zf^{\prime }\left(z\right)/g\left(z\right)\right)+\left(2\alpha -1\right)}\right|<\beta \;\left(z\in U;\;g\in MS^{*}\right), \end{array}$$

where (and throughout this paper unless otherwise mentioned) the parameters *α* and *β* are constrained as follows:

(16) $$\begin{array}{c} 0≦\alpha <1,\;0<\beta ≦1. \end{array}$$

It is easy to verify that *f* ∈ *MC*(*α*, *β*) if and only if

(17) $$\begin{array}{c} -\frac{zf^{\prime }\left(z\right)}{g\left(z\right)}≺\frac{1+\left(1-2\alpha \right)\beta z}{1-\beta z}\;\left(z\in U\right). \end{array}$$

We observe that

(18) $$\begin{array}{c} R\left(\frac{1+\left(1-2\alpha \right)\beta z}{1-\beta z}\right)>0\;\left(z\in U\right), \end{array}$$

and, thus, the function class *MC*(*α*, *β*) is a subclass of meromorphic close-to-convex functions.

Clearly, the class *MC*(*α*, 1) = :*MC*(*α*) is the familiar class of meromorphic close-to-convex functions of order *α*.

For some recent investigations of meromorphic functions, see, for example, the works of [7–22] and the references cited therein.

To derive our main results, we need the following lemmas.

**Lemma 2 (see [23]).**

Let

(19) $$\begin{array}{c} f_{1}\left(z\right)=1+\overset{\infty }{\underset{n=1}{\sum }}c_{n}z^{n} \end{array}$$

be analytic in *U* and let

(20) $$\begin{array}{c} f_{2}\left(z\right)=1+\overset{\infty }{\underset{n=1}{\sum }}d_{n}z^{n} \end{array}$$

be analytic and convex in *U*. If *f*_1_≺*f*_2_, then

(21) $$\begin{array}{c} \left|c_{n}\right|≦\left|d_{1}\right|\;\left(n\in N≔\left\{1,2,\ldots \right\}\right). \end{array}$$

**Lemma 3 (see [24]).**

Suppose that

(22) $$\begin{array}{c} h\left(z\right)=\frac{1}{z}+\overset{\infty }{\underset{n=1}{\sum }}c_{n}z^{n}\in MS^{*}. \end{array}$$

Then

(23) $$\begin{array}{c} \left|c_{n}\right|≦\frac{2}{n+1}\;\;\left(n\in N\right). \end{array}$$

Each of these inequalities is sharp, with the extremal function given by

(24) $$\begin{array}{c} h\left(z\right)=z^{-1}{\left(1+z^{n+1}\right)}^{2/\left(n+1\right)}. \end{array}$$

**Lemma 4 (see [25]).**

Let −1≦*D* < *C*≦1 and −1≦*F* < *E*≦1. Then,

(25) $$\begin{array}{c} \frac{1+Cz}{1+Dz}≺\frac{1+Ez}{1+Fz} \end{array}$$

if and only if

(26) $$\begin{array}{c} \left|ED-FC\right|≦\left(E-F\right)-\left(C-D\right). \end{array}$$

**Lemma 5 (see [26]).**

Suppose that the function **g** ∈ MS*. Then

(27) $$\begin{array}{c} \frac{{\left(1-r\right)}^{2}}{r}≦\left|g\left(z\right)\right|≦\frac{{\left(1+r\right)}^{2}}{r}\;\;\left(\left|z\right|=r;\;0<r<1\right). \end{array}$$

**Lemma 6 (see [27]).**

Suppose that

(28) $$\begin{array}{c} p\left(z\right)≺\frac{1+\left(1-2\alpha \right)z}{1-z}\;\left(z\in U;\;0≦\alpha <1\right). \end{array}$$

Then,

(29) R(zp′(z)p(z))≧{−2(1−α)r(1+r)[1+(2α−1)r](|z|=r; 0<r≦r0),(4α(1−r2)[1+(1−2α)r2] −[1+(1−2α)r2]) ×((1−α)(1−r2))−1 −α1−α(|z|=r; r0<r<1),

where *r*_0_ is the unique root of the equation

(30) $$\begin{array}{c} \left(2\alpha -1\right)r^{4}-2\left(2\alpha -1\right)r^{3}-6\alpha r^{2}-2r+1=0 \end{array}$$

in the interval (0,1). The results are sharp.

In the present paper, we aim at proving some coefficient inequalities, convolution property, inclusion relationship, distortion property, and radius of meromorphic convexity of the class *MC*(*α*, *β*).

## 2. Main Results

We begin by stating the following coefficient inequality of the class *MC*(*α*, *β*).

**Theorem 7 .**

Suppose that

(31) $$\begin{array}{c} f\left(z\right)=\frac{1}{z}+\overset{\infty }{\underset{n=1}{\sum }}a_{n}z^{n}\in MC\left(\alpha ,\beta \right). \end{array}$$

Then

(32) $$\begin{array}{c} \left|a_{1}\right|≦1, \end{array}$$

(33) |an|≦2(1−α)βn(1+∑k=1n−12k+1)+2n(n+1)(n∈N∖{1}).

**Proof**

Let *f* ∈ *MC*(*α*, *β*) and suppose that

(34) $$\begin{array}{c} p\left(z\right)∶=-\frac{zf^{\prime }\left(z\right)}{g\left(z\right)}\;\;\;\left(z\in U\right), \end{array}$$

where

(35) $$\begin{array}{c} g\left(z\right)=\frac{1}{z}+\overset{\infty }{\underset{n=1}{\sum }}b_{n}z^{n}\in MS^{*}. \end{array}$$

It follows that

(36) $$\begin{array}{c} p\left(z\right)=1+\overset{\infty }{\underset{n=1}{\sum }}p_{n}z^{n}≺\frac{1+\left(1-2\alpha \right)\beta z}{1-\beta z}\;\left(z\in U\right). \end{array}$$

In view of Lemma 2, we know that

(37) $$\begin{array}{c} \left|p_{n}\right|≦2\left(1-\alpha \right)\beta \;\left(n\in N\right). \end{array}$$

By substituting the series expressions of functions *f*, *g*, and *p* into (34), we get

(38) (1+p1z+p2z2+⋯+pnzn+pn+1zn+1+⋯)  ×(1z+b1z+b2z2+⋯+bnzn+⋯)   =1z−a1z−2a2z2−⋯−nanzn−⋯.

Since *f* is univalent in U*, it is well known that |*a*_1_|≦1.

On the other hand, we find from (38) that

(39) −nan=bn+p1bn−1+p2bn−2+⋯+pn−1b1+pn+1(n∈N∖{1}).

By noting that *g* ∈ MS*, it follows from Lemma 3 that

(40) $$\begin{array}{c} \left|b_{n}\right|≦\frac{2}{n+1}\;\left(n\in N\right). \end{array}$$

Combining (37), (39), and (40), we have

(41) n|an|≦2(1−α)β(2n+2n−1+⋯+23+1+1)+2n+1(n∈N∖{1}).

Thus, the assertion (33) of Theorem 7 follows directly from (41).

**Theorem 8 .**

Let

(42) $$\begin{array}{c} g\left(z\right)=\frac{1}{z}+\overset{\infty }{\underset{n=1}{\sum }}b_{n}z^{n}\in MS^{*}. \end{array}$$

If *f* ∈ Σ satisfies the condition

(43) $$\begin{array}{c} \left(1+\beta \right)\overset{\infty }{\underset{n=1}{\sum }}n\left|a_{n}\right|+\left(1+\left|2\alpha -1\right|\beta \right)\overset{\infty }{\underset{n=1}{\sum }}\left|b_{n}\right|≦2\left(1-\alpha \right)\beta , \end{array}$$

then *f* ∈ *MC*(*α*, *β*).

**Proof**

To prove *f* ∈ *MC*(*α*, *β*), it suffices to show that (15) holds. From (43), we know that

(44) β(2(1−α)−∑n=1∞n|an|−|2α−1|∑n=1∞|bn|)  ≧∑n=1∞n|an|+∑n=1∞|bn|>0.

Now, by the maximum modulus principle, we deduce from (1) and (44) that

(45) |(zf′(z)/g(z))+1(zf′(z)/g(z))+(2α−1)| =|∑n=1∞nanzn+1+∑n=1∞bnzn+1∑n=1∞nanzn+1+(2α−1)∑n=1∞bnzn+1+2(α−1)| <∑n=1∞n|an|+∑n=1∞|bn|2(1−α)−∑n=1∞n|an|−|2α−1|∑n=1∞|bn|≦β.

This evidently completes the proof of Theorem 8.

**Example 9 .**

By applying Theorem 8, it is obvious to see that the function

(46) $$\begin{array}{c} f\left(z\right)=\frac{1}{z}+z\in MC\left(0,1\right). \end{array}$$

**Theorem 10 .**

Let |*ξ*| = 1 and *g* ∈ MS*. A function *f* ∈ *MC*(*α*, *β*) if and only if

(47) f(z)∗[(1−βξ)1−2zz(1−z)2] −g(z)∗{[1+(1−2α)βξ]z2−z+1z(1−z)}≠0     (z∈U∗).

**Proof**

A function *f* ∈ *MC*(*α*, *β*) if and only if

(48) $$\begin{array}{c} -\frac{zf^{\prime }\left(z\right)}{g\left(z\right)}\neq \frac{1+\left(1-2\alpha \right)\beta \xi }{1-\beta \xi }\;\left(z\in U;\;\left|\xi \right|=1\right). \end{array}$$

It is easy to see that condition (48) can be written as

(49) zf′(z)(1−βξ)+g(z)[1+(1−2α)βξ]≠0(z∈U∗; |ξ|=1).

We observe that

(50) $$\begin{array}{c} -zf^{\prime }\left(z\right)=f\left(z\right)*\left(\frac{1}{z}-\frac{z}{{\left(1-z\right)}^{2}}\right)=f\left(z\right)*\frac{1-2z}{z{\left(1-z\right)}^{2}},\\ g\left(z\right)=g\left(z\right)*\left(\frac{1}{z}+\frac{z}{1-z}\right)=g\left(z\right)*\frac{z^{2}-z+1}{z\left(1-z\right)}. \end{array}$$

By substituting (50) into (49), we get the desired assertion (47) of Theorem 10.

**Theorem 11 .**

Let

(51) $$\begin{array}{c} 0≦\alpha_{2}≦\alpha_{1}≦\frac{1}{2},\;\;0<\beta_{1}≦\beta_{2}<1. \end{array}$$

Then,

(52) $$\begin{array}{c} MC\left(\alpha_{1},\beta_{1}\right)\subset MC\left(\alpha_{2},\beta_{2}\right). \end{array}$$

**Proof**

Suppose that *f* ∈ *MC*(*α*_1_, *β*_1_). We easily know that

(53) $$\begin{array}{c} -\frac{zf^{\prime }\left(z\right)}{g\left(z\right)}≺\frac{1+\left(1-2\alpha_{1}\right)\beta_{1}z}{1-\beta_{1}z}\;\left(z\in U\right). \end{array}$$

By setting *C* = 1 + (1 − 2*α*_1_)*β*_1_, *D* = −*β*_1_, *E* = 1 + (1 − 2*α*_2_)*β*_2_, and *F* = −*β*_2_, it follows from (51) that

(54) |ED−FC|  =|−(1−2α2)β1β2+(1−2α1)β1β2|  ≦|−(1−2α2)β1β2+(1−2α1)β12|  +|−(1−2α1)β12+(1−2α1)β1β2|  ≦[(1−2α2)β2−(1−2α1)β1]+(β2−β1)  =[(1−2α2)β2−(−β2)]  −[(1−2α1)β1−(−β1)]  =(E−F)−(C−D).

In view of Lemma 4, we deduce that

(55) −zf′(z)g(z)≺1+(1−2α1)β1z1−β1z≺1+(1−2α2)β2z1−β2z(z∈U),

which implies that *f* ∈ *MC*(*α*_2_, *β*_2_). Thus, the assertion (52) of Theorem 11 holds.

**Theorem 12 .**

Let *f* ∈ *MC*(*α*, *β*). Then,

(56) (1−r)2[1−(1−2α)βr]r2(1+βr) ≦|f′(z)|≦(1+r)2[1+(1−2α)βr]r2(1−βr) (|z|=r;   0<r<1).

**Proof**

Let *f* ∈ *MC*(*α*, *β*). By definition, we know that

(57) $$\begin{array}{c} -\frac{zf^{\prime }\left(z\right)}{g\left(z\right)}≺\frac{1+\left(1-2\alpha \right)\beta z}{1-\beta z}\;\;\;\;\;\;\;\;\left(z\in U\right). \end{array}$$

Suppose that the function *p* is defined by (36). Then, we have

(58) 1−(1−2α)βr1+βr≦|p(z)|≦1+(1−2α)βr1−βr, (|z|=r; 0<r<1).

Since *g* ∈ MS*, by Lemma 5, we know that

(59) $$\begin{array}{c} \frac{{\left(1-r\right)}^{2}}{r}≦\left|g\left(z\right)\right|≦\frac{{\left(1+r\right)}^{2}}{r}\;\left(\left|z\right|=r;\;0<r<1\right). \end{array}$$

Thus, by virtue of (36), (58), and (59), we readily get the assertion (56) of Theorem 12.

Finally, we derive the radius of meromorphic convexity for the class *MC*(*α*).

**Theorem 13 .**

Let *f* ∈ *MC*(*α*) with 0 < *α* < 1. Then,

1. for *r*_1_≦*r*_0_, *f* is meromorphic convex in 0 < |*z*| < *r*_1_;
2. for *r*_1_ > *r*_0_, *f* is meromorphic convex in 0 < |*z*| < *r*_2_,

where *r*_0_ is the unique root of the equation

(60) $$\begin{array}{c} \left(2\alpha -1\right)r^{4}-2\left(2\alpha -1\right)r^{3}-6\alpha r^{2}-2r+1=0 \end{array}$$

in the interval (0,1) and *r*_1_ and *r*_2_ are the smallest root of the equations

(61) $$\begin{array}{c} \left(2\alpha -1\right)r^{3}+3\left(2\alpha -1\right)r^{2}+3r+1=0,\\ \left(1-2\alpha \right)r^{4}+2\left(1-2\alpha \right)r^{3}+3\left(1-\alpha \right)r^{2}+2r+1=0 \end{array}$$

in the interval (0,1), respectively.

**Proof**

Let *f* ∈ *MC*(*α*) and suppose that

(62) $$\begin{array}{c} q\left(z\right)∶=-\frac{zf^{\prime }\left(z\right)}{g\left(z\right)}\;\;\left(z\in U\right). \end{array}$$

Then,

(63) $$\begin{array}{c} q\left(z\right)≺\frac{1+\left(1-2\alpha \right)z}{1-z}\;\;\left(z\in U\right). \end{array}$$

It follows from (62) that

(64) $$\begin{array}{c} -zf^{\prime }\left(z\right)=q\left(z\right)g\left(z\right). \end{array}$$

Differentiating both sides of (64) logarithmically, we get

(65) $$\begin{array}{c} -\left(1+\frac{zf^{\prime \prime }\left(z\right)}{f^{\prime }\left(z\right)}\right)=\frac{zq^{\prime }\left(z\right)}{q\left(z\right)}+\frac{zg^{\prime }\left(z\right)}{g\left(z\right)}. \end{array}$$

Since *g* ∈ MS*, we know that

(66) $$\begin{array}{c} R\left(\frac{zg^{\prime }\left(z\right)}{g\left(z\right)}\right)≧-\frac{1+r}{1-r}\;\left(\left|z\right|=r\right). \end{array}$$

Combining (63), (65), (66), and Lemma 6, we obtain

(67) −R(1+zf′′(z)f′(z))≧{−1+r1−r −2(1−α)r(1+r)[1+(2α−1)r]=:H~α(r)(|z|=r; 0<r≦r0),−1+r1−r +(4α(1−r2)[1+(1−2α)r2]  −[1+(1−2α)r2])  ×((1−α)(1−r2))−1 −α1−α=:F~α(r)(|z|=r; r0<r<1),

where *r*_0_ is the unique root of (30) in the interval (0,1). It follows from (67) that the bound of meromorphic convexity for the class *MC*(*α*) is determined either by the equation

(68) $$\begin{array}{c} -\frac{1+r}{1-r}-\frac{2\left(1-\alpha \right)r}{\left(1+r\right)\left[1+\left(2\alpha -1\right)r\right]}=0, \end{array}$$

or by the equation

(69) −1+r1−r +4α(1−r2)[1+(1−2α)r2]−[1+(1−2α)r2](1−α)(1−r2) −α1−α=0.

We note that (68) and (69) can be rewritten as follows:

(70) Hα(r)∶=(2α−1)r3+3(2α−1)r2+3r+1=0,Fα(r) ∶=(1−2α)r4+2(1−2α)r3+3(1−α)r2+2r+1   =0.

Let *r*_1_ and *r*_2_ be the smallest root of the equations *H*_*α*_(*r*) = 0 and *F*_*α*_(*r*) = 0 in the interval (0,1), respectively. By observing that *H*_*α*_(0) = 1 > 0, we deduce that *H*_*α*_(*r*) > 0 for *r* < *r*_1_.

Similarly, we know that *F*_*α*_(*r*) > 0 for *r* < *r*_2_, since *F*_*α*_(0) = 1 > 0.

We observe that

(71) $$\begin{array}{c} {\tilde{H}}_{\alpha }\left(r\right)≧0⟺H_{\alpha }\left(r\right)≧0,\\ {\tilde{F}}_{\alpha }\left(r\right)≧0⟺F_{\alpha }\left(r\right)≧0. \end{array}$$

Thus, when *r*_1_≦*r*_0_, *f* is meromorphic convex in 0 < |*z*| < *r*_1_; when *r*_1_ > *r*_0_, *f* is meromorphic convex in 0 < |*z*| < *r*_2_.

The proof of Theorem 13 is thus completed.
